# How can the education sector support children’s mental health? Views of Australian healthcare clinicians

**DOI:** 10.1371/journal.pone.0261827

**Published:** 2022-01-24

**Authors:** Kate Paton, Lynn Gillam, Hayley Warren, Melissa Mulraney, David Coghill, Daryl Efron, Michael Sawyer, Harriet Hiscock

**Affiliations:** 1 Centre for Community Child Health, Murdoch Children’s Research Institute, Melbourne, Victoria, Australia; 2 Melbourne School of Population and Global Health, The University of Melbourne, Melbourne, Victoria, Australia; 3 Children’s Bioethics Centre, The Royal Children’s Hospital, Melbourne, Victoria, Australia; 4 ISN Innovations, Institute for Social Neuroscience, Ivanhoe, Victoria, Australia; 5 Department of Paediatrics, University of Melbourne, Melbourne, Victoria, Australia; 6 Mental Health, The Royal Children’s Hospital, Melbourne, Victoria, Australia; 7 Department of Psychiatry, University of Melbourne, Melbourne, Victoria, Australia; 8 School of Medicine, University of Adelaide, Adelaide, South Australia, Australia; 9 Research and Evaluation Unit, Women’s and Children’s Health Network, North Adelaide, South Australia, Australia; 10 Health Services Research Unit, The Royal Children’s Hospital, Melbourne, Victoria, Australia; King Saud University, SAUDI ARABIA

## Abstract

**Objectives:**

Policy makers in developed countries have long considered the education system an avenue for supporting mental health care for children. Whilst educators have identified many challenges to providing this support (e.g. non-core role, stigma, overcrowded curriculum), understanding clinicians’ views on the role of educators and schools and how clinicians and schools could work together to achieve good mental health outcomes are important questions. However, clinician voices in how schools and health should work together for children’s mental health care are frequently missing from the debate. We aimed to report clinicians’ views about how the education system could support student’s mental health and improve access to mental health care for children and adolescents.

**Methods:**

143 clinicians (approximately 35 each of child and adolescent psychiatrists, pediatricians, child psychologists and general practitioners (GPs)) from the states of Victoria and South Australia participated in semi-structured phone interviews between March 2018 and February 2019. Inductive content analysis was applied to address the broad study aims.

**Findings:**

Key themes emerged: (1) The role of schools in supporting individual children; (2) School based programs to support children and families; and (3) Challenges of implementing these suggestions. Clinicians across all professional groups suggested the education system could play an important role in improving access to mental health services through harnessing existing staff or co-locating mental health clinicians. They also suggested schools could identify at risk children and implement coping and social skills programs.

**Conclusions:**

Schools and educators could play a key role in prevention and early intervention of children’s mental health problems. However, before recommending exactly how to do this, key evidence gaps need to be addressed.

## Introduction

Reducing the prevalence of common child mental health conditions is a challenge for governments and policy makers. Despite reports identifying necessary system level changes over the last three decades [1–6], the prevalence of mental health conditions such as anxiety and depression, which are the major source of disease burden, has remained unchanged for the past 20 years [7, 8]. With approximately 50% of mental health disorders beginning before the age of 14 years [9], prevention and early intervention are paramount if we want to reduce lifetime prevalence of mental health disorders and allow children to live their best possible lives. Improving mental health for children and adolescents has therefore become an international priority [10].

Children spend a large proportion of their waking hours at school. Thus, education systems have been a focus of policy makers for prevention and early intervention for mental health problems [4, 5, 11–13]. Primary prevention can be defined as interventions that take place before the initial onset of a mental health problem to prevent the problem developing, whilst early intervention can be defined as interventions for individuals who are displaying the early signs and symptoms of a mental health problem or experiencing a first episode of a mental health problem [14]. In the UK, the National Institute for Health and Care Excellence and the Commission on Children and Young People’s Mental Health expect educators to be able to identify early signs of mental health problems in students and provide early intervention for mental health problems [15, 16]. In Australia, a national government-funded initiative called Be You is providing online training to educators to help them identify signs and symptoms of mental health problems in students but does not call for educators to provide early intervention [17]. Further, the evidence base for this initiative is modest [18].

Internationally, there is considerable debate as to whether educators should be asked to identify early signs of mental health problems and whether they should be asked to provide early intervention [19–21]. This is in part because educators are increasingly being asked to do more for many issues within a crowded curriculum (e.g. reduce obesity, manage oral health, foster healthy relationships [22–24] whilst at the same time, educate children to ensure they reach their academic potential. However, mental health problems have such a large adverse effect on children’s education progress that academic potential cannot be achieved unless schools address student mental health [25]. There is also concern that parents may not want educators to identify mental health problems in their child. However, a recent UK study of 290 parents suggests that screening for mental health difficulties would be acceptable to 82%, with the minority concerned about stigma towards students, inaccurate identification and low uptake of services once identified [26].

Although policy makers and researchers have identified schools as a key component of any system level improvement, studies of educators’ perspectives (i.e. teachers and school principals) have identified major challenges in supporting students’ mental health needs [27–32]. These challenges include communication with health professionals and confidentiality, time constraints for teachers, poor school leadership (particularly in student mental health), lack of teacher training and competence in detecting mental health problems, and perceived stigma [27, 28, 30]. Many educators also feel frustrated about overburdened specialist health services, which often leave teachers to address children’s mental health needs as they wait for an appointment with a health professional [27].

While educators have experience in education systems, and policy makers view education systems as a mechanism for improving access to mental health clinicians for children and adolescents, it is also important to understand whether different perspectives may exist between educators and mental health clinicians which need to be bridged if these professionals are to work successfully together to achieve both good education and mental health outcomes. However, clinician voices in how schools and health should work together for children’s mental health care are frequently missing from the debate. In Australia, most mental health care is provided by clinicians based in public facilities (in hospital clinics) or private practice and these clinicians include child and adolescent psychiatrists, psychologists and pediatricians. General practitioners (GPs) in the community also provide a large amount of care and are the key referrers to specialist mental health care. A smaller group of clinicians work out of schools, usually comprising psychologists and counsellors [8]. A recent study of clinicians who typically interact with schools—i.e. child and adolescent psychiatrists, psychologists, pediatricians and GP’s- when asked about system level reform reported education systems as an important potential mechanism for improving mental health outcomes for children and adolescents [33]. This echoes our previous study focusing on ADHD and Autism [34]. However, rigorous examination of clinician perspectives on *how* this should happen is lacking.

The original aim of the current study was to focus on clinician views about the efficacy of the healthcare system broadly. However, as this paper reports, a rigorous qualitative analysis of the data identified that the education system and its role in supporting mental health was a consistent theme across all clinician groups. Therefore, the aim of this paper is to report clinicians’ views about how the education system could support student’s mental health and improve access to mental health care for children and adolescents.

## Methods

This qualitative study was nested within a larger study “Towards an equitable mental health system for children and adolescents”, investigating barriers, enablers and potential opportunities for system level changes to improve equitable access to mental health services for Australian children and adolescents with common mental health conditions. These conditions included Attention Deficit Hyperactivity disorder (ADHD), anxiety and depression, which, over a 12 month period, affect 7.8%, 6.9% and 3.2% of Australian children aged 4–17 years, respectively [8]. The findings reported in this paper reflect a subset of the overall findings from this larger qualitative study.

### Recruitment

A purposive sampling strategy was used to recruit child and adolescent psychiatrists, pediatricians, child psychologists and GPs in two states of Australia (Victoria and South Australia) working in both low and high socioeconomic areas, metropolitan and regional areas, and public (where clinicians ‘bulk bill’- i.e. charge no out of pocket costs to the patient for a service) and private clinical settings.

Participants were sourced via 3 key strategies:

- Websites linked to professional organisations, e.g. Find a Psychiatrist [35]
- Key informants [36] from within professional networks of pediatricians, child and adolescent psychiatrists, psychologists and GPs,
- Internet searches covering the first 5 results pages on Google for each clinician group, by state (i.e. search terms “clinician, location”).

Where these recruitment strategies combined generated lists larger than 20 clinicians (for each clinician group), a statistician not associated with the project assigned a random number to each clinician and the clinicians were contacted in order of the numerical number assigned.

### Procedures

Research Assistants with no clinical background (KP, HW and SR) conducted 143 semi-structured phone interviews with clinicians (35 pediatricians, 35 child and adolescent psychiatrists, 37 psychologists and 36 GPs) between March 2018 and February 2019.

Approval was received from Human Research Ethics Committee at The Royal Children’s Hospital, Melbourne, Victoria, Australia (HREC 37105). Prior to interviews, participants were informed that their participation was voluntary and unpaid. Verbal informed consent was sought.

Participants were provided with one of two alternately allocated vignettes (either a child with ADHD or anxiety symptoms, (S1 and S2 Appendices) prior to the interview. This was designed to focus them on common child mental health conditions. Interviews lasted approximately 45 minutes. A semi-structured interview guide (S3 Appendix) was used to ensure some key themes were covered, but also allowed participants to discuss what was important to them.

Detailed field notes were kept for all interviews. Reflexivity was maintained by ongoing discussions between researchers and reflexivity journals. Interviews were audio recorded and transcribed verbatim. Transcripts were validated, de-identified and participants assigned pseudonyms. Transcripts were then coded for analysis using NVIVO 12.0 [37] software programme.

### Analyses

Categories and themes were identified using the processes of content analysis [38]. Three researchers (KP, HW and SR) each coded 4 transcripts separately to identify emerging themes. These themes (and all transcripts) were discussed with and reviewed by an experienced qualitative researcher (LG). Following this, an initial coding schema was developed and agreed by these 4 researchers. The coding schema was discussed with the Principal Investigator (HH) and analysis proceeded using this coding schema. Field notes collected during interviews were reviewed to seek clarification as appropriate. Regular discussion between members of the research team ensured a rigorous process of qualitative coding to identify similarities and differences, enable iterative development and validation of emergent themes. Transcripts from each clinician group were coded separately. Analysis continued until data saturation was achieved in relation to the research question which was after approximately 10 transcripts from each clinician group were coded.

A further 8 transcripts were then reviewed for each clinician type to ensure representation across the sample and to validate the themes. Results are reported from analysis of 72 interviews. Findings are reported in line with the COnsolidated criteria for REporting Qualitative research (COREQ) [39] checklist.

## Results

### Demographics

Workplace characteristics of participating clinicians are shown in Table 1. There was a spread of clinician types across type of practice and socioeconomic settings. More respondents practised in metropolitan than rural/remote settings and more psychologists practised in private than public settings, likely reflecting workforce distributions.

**Table 1:** Clinician characteristics by work settings.

	Location*		Socioeconomic status*			Health setting		
	Regional/remote (N)	Major city (N)	Low (N)	Medium (N)	High (N)	Private only (N)	Public only (N)	Public and Private (N)
Psychiatrist	14	28	32	30	27	7	10	18
Psychologist	13	26	25	21	29	31	1	5
Paediatrician	12	28	33	32	25	5	10	20
General practitioner	10	28	27	29	21	16	2	18

* Several clinicians practice in both regional/remote settings and major city settings and across socioeconomic areas

### Findings

Analysis of the interview transcripts from a broad range of participants revealed important themes which are presented using verbatim quotes. Some quotes have been truncated without changing the meaning. This is represented by an ellipsis (…..).

The overall study identified 5 overarching key themes. In this paper we provide a summary of findings from one key theme: the importance of the education sector in mental health for children and adolescents and identifying an evidence-based role for that sector. Other themes in the broader study included: health sector challenges and components of an optimal system for child and adolescent mental health care; health system funding models; the role of emotion and perceptions about mental health; and supporting parents to support their child.

### Beneficial role of the education system in accessing mental health services for children and adolescents

Clinicians frequently identified that improving access to care could not be resolved by changes to the health system alone. Many clinicians across all professional groups identified that the education system could play an important role in improving access to mental health services. They also expressed a desire for the health and education systems to work together to improve outcomes. Three key themes emerged: (1) The role of schools in supporting individual children; (2) Programs within the school environment to support children’s coping and mental health literacy and (3) Challenges of implementing these suggestions.

### (1) The role of schools in supporting individual children

#### An accessible place for support to be offered

Given that school attendance is a requirement and schools are typically located close to home, services provided at schools may be easier for students to access.

*Cause you know what…a lot of kids*, *they’re probably happy to see us but they often don’t want to come*, *because it might be their parents’ practice or they worry initially that we may discuss things with their parents*, *they don’t know about confidentiality*. *So by putting it in a neutral place like a school*, *I think that avoids a lot of those situations*. (Victorian GP 2, private)

*Most large high schools do have a nurse*, *and if that nurse had been comfortable to also be having to be involved in the management of the anxious*, *the depressed*, *the self-harming*, *the substance abusing adolescent*, *then that may well work quite well*, *because that nurse is then part of the school itself*, *part of the fabric of the school*. (Victorian GP 3, private/bulk billing)

#### Schools are physically located close to families

Clinicians identified that services close to home would assist access to attend the appointments in person and this may also be an area where schools could have a role.

*But certainly close to the schools would be good*, *so that they don’t have to travel too far*. (Victorian GP 19, private only)

*There just needs to be equity of access*. *And there needs to be local access*. *There needs to be you know local facilities that can see these children*. *And sometimes local means not within health*, *you know not to come to a hospital or a community health centre*, *it might be that it needs to be in schools* (Victorian Pediatrician 28, public only)

#### Working as a team with health care professionals

Clinicians identified the school setting as an opportunity to support students and families by working together.

*I actually think school should be so much more than that*, [getting an ATAR (Australian Tertiary Admission Rank–for entry to tertiary education*], *if it was able to do more than that it would like really improve health*, *and so I think if we could consult to schools as mental health professionals and provide them with support structures*, *and help them to set up programs that would be really helpful*. (Victorian Child & Adolescent Psychiatrist 13, private only)

*So it’s not just a matter of seeing a child and diagnosis and treating*. *It’s all about designing a package for supporting families*. *…*. *We have a nought to twelve program for kids that are falling out of school*, *so we can get at them early to stop the school refusal*. *Because often the best medicine is school*. *(*Victorian Child & Adolescent Psychiatrist 31, public only)

Formalised communication mechanisms which meant that health care professionals could work more closely with educators and support staff were highlighted as an important component to a collaborative system.

*I think they’re also quite reluctant to approach GPs*. *They’re always surprised that*, *you know*, *if I make a phone call or I request something*, *"Oh*, *you want to be involved*?*" Or*, *"You’ve actually bothered to ring me*?*" you know*, *they’re surprised that we could work as a team*. *And that would make perfect sense to me*, *if we could have some service through the schools*, *would be good too*, *where we could have good communication and work together*. (Victorian GP 19, private only)

School-based services could be used as a more efficient means for students accessing treatment but not wanting to miss too much school due to a lack of availability of after-hours appointments.

*The other sorts of barriers I see with children*, *and particularly adolescents*, *is missing school time*. *You know they want after-hours therapy and I don’t really offer after-hours therapy*. (South Australian Psychologist 1, private only)

#### Monitoring student attendance and academic performance

Some clinicians felt that schools are well placed to identify students with mental health problems as systems exist within the education sector for monitoring. For example, monitoring attendance records can track students that may miss school because of underlying mental health problems.

*kids with moderate or severe depression miss on average 40 days a year*. *…*..*could that be a flag to say hey this kid’s struggling*, *let’s just look at why that is and could this represent a mental health issue and let’s see if we can review that*. *And then track progress with simple measures already being done like attendance*. *(*Victorian GP 31, bulk billing/private)

Or by considering (lack of) academic progress as an opportunity to engage with a child or adolescent to monitor their mental health.

*So I said to him*, *in a sense of hoping to engage him*, *"Oh look*, *I’m sorry*, *to hear your grades have gone down*, *and is that a concern to you” …*..*“if you do feel like you want to engage in support at this time*, *that’s fine*, *that is your choice*, *but I’d love to kind of just see how things go with your grades*. *And is it okay if we just kind of monitor that*? *And maybe if things aren’t kinda going well*, *it might indicate that you a need a little bit more support and we’d like to help you out*.*”…*… *And he said that was fine*, *we could do that*. (Victorian GP 31, bulk billing/private)

### (2) Programs within the school environment to support children’s coping and mental health literacy

#### Upskilling students through programs

Some clinicians suggested school-based skill building programs e.g. coping skills could be valuable in prevention of mental health conditions.

*the other thing I’ve always thought would be very helpful in schools is a kind of psycho-ed program that you run in schools so that*, *all the kids get the same information about how to manage anxiety*. *You know*, *what’s a sign of something wrong*? *How to manage friendships*, *that’s a big issue for kids in school*. *Just giving them coping skills because a lot of what we do on an individual basis could be done as a group*. (Victorian Psychologist 7, private only)

*And if there were more programs to put*, *support social interaction and stuff like that in a way that wasn’t intrusive*, *I think that that would really help young people*. (Vic Child & Adolescent Psychiatrist 13, private only)

*So in China for example*, *they have*, *mental health teachers*. *So they have teachers who teach a subject around mental health that teaches kids from grade one onwards how to identify the difference between thoughts and feelings and emotions*, *why emotions are important*. *So it’s all that basic mental health literacy stuff*. *Where to get support*, *how to build resilience*, *how to challenge anxiety*. (Victorian Psychologist 16, public/private)

Interventions designed to address bullying were highlighted.

*you know I guess bullying*, *that’s an ongoing epidemic that is part and parcel unfortunately of the human condition but better programs or*, *again*, *more funding to tackle bullying in schools and to improve social cohesion would be excellent*. *That would certainly minimise the number of children that end up on my doorstep*. (South Australian Psychologist 3, private only)

#### Supporting social connectedness

Some clinicians suggested that schools can play a role in developing the student’s connectedness through extra-curricular activities, which could improve mental health indirectly.

*Having*, *say*, *programs at lunchtime maybe in a school where there was a range of different areas of interest that*, *where there were a range of different areas that kids could go to and link in with other people with similar interest groups*,…… (Victorian Child & Adolescent Psychiatrist 13, private only)

*there’s a lot of psycho-social spill over*. *A lot of violence*, *a lot of family problems*. *And a lot of that manifests itself as symptoms*. *… maybe let’s just do what we know to help kids’ mental health*. *You know and making them all play football*. *You know*, *something more sensible…*. (Victorian Child & Adolescent Psychiatrist 31, public only)

#### Upskilling educators

Peer support for school psychologists was suggested as well as mental health literacy education for teachers.

*…*.*but sometimes these kids are really challenging in the classroom*, *and I think for teachers*, *one of the really important things that we can do as pediatricians is actually provide education to teachers*, *and reframe and rephrase the child’s behavior based on whatever is going on for that child*. (South Australian pediatrician 1, public only)

….. *when I did my training in my master’s*, *we did a component on community psychology*. *you know this sort of preventive type of thing*, *and I would love to be able to give consultation services for example*, *to schools…*.. *Or like*, *run a group with school psychologists … like a weekly peer discussion group*. *(*Victorian Psychologist 2, private only)

### (3) Challenges to involving schools in mental health services access

Although clinicians found many benefits in the involvement of the education sector, they also found several challenges, as outlined below.

School services stop during school holidays and this was seen by clinicians as disruption to therapeutic benefits.

*So*, *there’s a lot of to-ing and fro-ing between myself and the psychologist at the school*, *so*, *it works great when they’re there*, *and they’re able to catch up with them on a weekly or fortnightly basis*. *But then*, *there’s this big handover process that happens every semester for me to catch up with them when they’re away*….. *when the psychologist isn’t there*. (Victorian GP 4, private only)

School leadership determined the strength of response to mental health strategies.

*But it has been difficult to replicate in other high schools*. *But I think that is a good model*, *that works well*, *but it only works well at [xxx] because the headmaster is very driven by the needs of his students* (Victorian GP 3, public /private)

Others felt that school is not an appropriate place for students to be seeing a health professional due to stigma associated with being taken out of class as well as privacy issues.

*I think there are issues with it being in schools*, *because I think everybody knows who goes to the see the school counsellor*, *and young people often don’t like that*. (Victorian Pediatrician 6, private/public)

Schools have different levels of resourcing for mental health support.

*We find that particularly depending on the school as well*, *the Catholic education system seem more inclined to fund additional assessments and therapy at school…*..*whereas the independent private schools often expect the families to fund that from their own pocket…*..*whereas the public schools*, *while they’ll pay for it*, *there’s often a very very long waiting list*. (SA Child & Adolescent Psychiatrist 5, public/private)

Clinicians also felt that there was inconsistency in educator’s mental health literacy.

…. *It’s not their training*, *so I’m not criticizing them*. *But I don’t think a lot of schools recognize the impact of anxiety on children*. *Their behavior that manifests because of their anxieties is punished or reprimanded or what have you*, *without recognition that it’s provoked by anxiety*, *without education*. (Victorian Pediatrician 14, public/private)

*Still unfortunately we get a lot of teachers who are very dismissive of mental health*, *…*..*So the person might go off early to see a therapist or they might see a psychologist at school or a counsellor at school*. *That’s often minimised and discredited saying often*, *“oh you know I don’t agree with that diagnosis” or “you don’t need that*,*” “you just need to toughen up”…*. *I think it then goes back to level or lack of training of teachers* (South Australian Child & Adolescent Psychiatrist 8, public/private)

#### Contribution of school culture to mental health

Clinicians suggested that genuine mental health support was not considered a priority in some schools and rather lip service was paid to supporting children and families.

*I see children from about six or seven different schools in this area*, *and it’s hard for me to keep track of which school has what*, *but I do get a general feel that some kids that are sort of quite quickly referred to an in house counselor or psychologist*, *and then they might get referred on to me or other people*. *So there seems to be a fairly well entrenched process there for dealing with problems*, *whereas …*…. *the issues may not be getting picked up or identified as well as they could be*, *depending on what the resources are at each school*. (Vic GP 15, public/private)

Some clinicians felt that the school system itself was a contributor to the growth of mental health issues. This was largely due to a structured approach that does not acknowledge needs of individual children.

*…*..*The education system needs to cater for the wider group of kids and it really needs to do so for the ADHD kids*, *for the kids on the spectrum that are high functioning*. *This is the majority of the kids that I see that are having mental health issues*, *it’s because school’s a large part of it*. (Vic Psychologist 1, private only)

## Discussion

This study has examined, for the first time, Australian clinician perspectives on the role of school and educators in improving child mental health. We could find no other published papers on clinicians’ views of how the education system can support children’s mental health. Clinicians largely viewed schools as a neutral place to offer support close to the family home. Clinicians often believed schools can offer prevention by monitoring students at risk of poor mental health (e.g. through attendance data) and supporting children through school wide psychoeducation programs, sport, and specific programs such as social skills and coping programs. Schools as buildings could also act as a trusted physical space where mental health clinicians could offer services that are otherwise challenging to access. Many clinicians were keen to reach out to schools to offer services or their professional support to school-based mental health staff, but this would likely require additional funding to do so. Whilst some research has focused on perspectives of clinicians working *within* an education setting, our study details perspectives of clinicians working *outside* the education setting, which is the norm for most health professionals in Australia.

Based on the views of Australian clinicians who care for children with mental health problems, schools and educators could play a key role in prevention and early intervention of children’s mental health problems. However, before recommending exactly how to do this, key evidence gaps need to be addressed. These include a lack of rigorous trials to determine: (i) the effectiveness and cost-effectiveness of screening for mental health problems versus monitoring children with established problems and associated outcomes including uptake of services [10] and longer term impacts on mental health; (ii) relative effectiveness and costs of universal versus targeted intervention programs; and (iii) long-term effectiveness and costs of school-wide programs that aim to foster resilience and coping skills. Many Australian schools do offer skills-based programs for children that aim to build their resilience and foster problem solving and positive social interactions, in an attempt to prevent mental health disorders. However, a 2018 review of such programs in primary schools found that they may be effective, if delivered by teachers, but few were evaluated by randomized controlled trials and long-term effects were limited [40, 41].

Many clinicians in this study also called for more effective ways to upskill educators in prevention, identification and early intervention for children’s mental health, including providing professional supervision for educators. The Australian government has funded an online intitaive for educators–Be You–with knowledge, resources and strategies to enable educators to “help children achieve their best possible mental health” [17]. Whether this leads to changes in educator’s ability to detect and respond mental health problems needs to be evaluated. Telementoring models delivered in healthcare such as Project ECHO [42] could be adapted and trialed for this purpose, especially in a post Covid-period where people have become more familiar with using online platforms to communicate. Project ECHO provides regular telementoring, in a format that begins with education about a particular health topic, delivered by an expert in that topic. This is followed by a case study presented by a non-expert. The telementoring group then disucsses the case including management options, with all attendees learning from each others’ areas of expertise. This model could be used in health-education collaborations across schools with the child mental health expert providing didactic teaching followed by an educator presenting a case. Other models include upskilling an educator at each school to better identify and refer children with mental health difficulites to local services. This model is being piloted by the state government of Victoria, Australia but again will require data to establish its effectivness and cost-effectiveness [43].

This study has a number of strengths. We interviewed a broad range of clinicians working across a variety of socioeconomic, geographical and practice settings. However, it is important to acknowledge the limitations of conducting qualitative research. The aim of this study was not to generalize findings to the population, but to document and perceptions and meaningful experiences with applications for policy, practice, and future research. Participants expressed an interest in discussing their experiences and were willing to give up their time to participate in the research. Findings are restricted to English speaking clinicians. Most professionals interviewed worked in metropolitan settings, reflecting the distribution of the workforce, but possibly reducing the breadth of insights from clinicians practising in rural settings. This paper reports on only one perspective (that of clinicians) which needs to be married with the perspective of others in the education sector to achieve consensus and good teamwork.

## Conclusions

In summary, this is the first Australian (and to the best of our knowledge, international) study to examine clinician perspectives on how the education sector can support prevention and early intervention for children’s mental health. Clinicians have voiced a range of ideas which can now be trialled and evaluated as programs to inform evidence-based practice and policy and in turn strengthen care for children’s mental health in education settings.

## Supporting information

S1 Appendix Vignette A ADHD. (DOCX)

S2 Appendix Vignette B anxiety. (DOCX)

S3 Appendix Interview guide. (DOCX)
