# Supplementary material for: How can the education sector support children’s mental health? Views of Australian healthcare clinicians
Source: PLoS One. 2022 Jan 24;17(1):e0261827. doi: 10.1371/journal.pone.0261827 (PMC8786182; doi:10.1371/journal.pone.0261827)
Supplement: S1 Appendix — (DOCX) [file pone.0261827.s001.docx]

S1 Appendix Vignette A ADHD

***Vignette A – Cameron***

*Cameron is an 8-year-old boy living with his mother and older brother. Cameron’s behavioural problems began 3 years ago, roughly around the time that his father moved out of their family home. Now at primary school, his mother is concerned about his persistent academic difficulties and behavioural problems. While Cameron works well when supervised individually and doesn’t bully other children, his teacher reports that he loses focus, loses or forgets his homework, and distracts his classmates. Cameron has a number of friends but also reports being teased for misbehaving. His mother finds Cameron’s defiant behaviour at home difficult, reporting that he persistently disobeys her repeated requests to get dressed or put his belongings away.*
