# Supplementary material for: How can the education sector support children’s mental health? Views of Australian healthcare clinicians
Source: PLoS One. 2022 Jan 24;17(1):e0261827. doi: 10.1371/journal.pone.0261827 (PMC8786182; doi:10.1371/journal.pone.0261827)
Supplement: S2 Appendix — (DOCX) [file pone.0261827.s002.docx]

S2. Appendix - Vignette B Anxiety

***Vignette B – Anna***

*Anna is a 13-year-old girl and eldest of 3 children in a close family. Her mother reports her to have always been a quiet, nervous child. Through primary school, Anna performed well academically but struggled to make friends. Since she started high school 6 weeks ago, she has complained of abdominal pain in the morning at least three times a week and as a consequence missed several days of school. She has avoided visiting other children’s houses, school camps and excursions because she doesn’t like being away from home. Anna checks her lunchbox every morning before leaving for school to check that her mum has packed her the same food every day, and if something is different she will be too upset to leave the house. Her mother also reports that Anna has difficulty sleeping and frequently asks her parents for reassurance when going to sleep and often multiple times through the night.*
