# Supplementary material for: How can the education sector support children’s mental health? Views of Australian healthcare clinicians
Source: PLoS One. 2022 Jan 24;17(1):e0261827. doi: 10.1371/journal.pone.0261827 (PMC8786182; doi:10.1371/journal.pone.0261827)
Supplement: S3 Appendix — (DOCX) [file pone.0261827.s003.docx]

S3 Appendix - Interview guide

**Interview Guide: Health Practitioners**

**Name: _______________________________________ Phone number: ______________________________**

**Study ID: _____________________________________ Date & Time: ________________________________**

**Profession: ___________________________________ Vignette: ___________________________________**

**State: _______________________________________ Interviewer: ________________________________**

**Setting: __________________________________________________________________________________________**

**__________________________________________________________________________________________________________________________________________________________________________________________________**

**SECTION 1 – INDIVIDUAL USUAL PRACTICE IN CURRENT SYSTEM**

| **Question** | **Checklist – ask if not answered in questions and/or if applicable** | **Question complete** |
| --- | --- | --- |
| 1. Imagine *<Cameron/Anna>* presented to you. Could you talk me through what you would normally do in your first consult? | - Are there any guidelines that you would use during your consult? - *[If they use guidelines]* Which guidelines do you find helpful [or use]? - Would you involve anyone else at this point? - Would you do any tests or questionnaires? |  |
| 1. If *<Cameron/Anna>* met criteria for *<ADHD/anxiety>*, what would you normally do in subsequent consults? 2. Is there a situation where you would refer *[Cameron/Anna]* elsewhere? 3. If you did refer    1. How do you decide who to refer to? 4. *[If applicable]* Would any of this be different in your private/public work? | - Would you consider a trial of medication?   - At what point?   - *[If no]* what would need to be different for you to consider medication? - Would you consider counselling?   - At what point?   - *[If no]* what would need to be different for you to consider counselling? - *[If they don’t refer]* What would need to be different for you to refer? - Would you take into account the out of pocket costs of the providers you refer to? - What if there’s a long waiting list? |  |
| 1. Within the current system, what do you see as the challenges to providing best care? 2. What practical changes would help you be more effective in delivering care to children and adolescents? | - Some people have suggested that the issue of stigma is still a challenge. Do you have any thoughts? - What role do you see for a GP / other health practitioner? |  |

**SECTION 2 – IMAGINING PRACTICE IN A FUTURE SYSTEM**

*[So we’ve been discussing practicing in the current health system. Now I’d like to expand out of this current system and move on to imagine/talk about what an ideal system or world would look like]*

| **Question** | **Checklist – ask if not answered in questions and/or if applicable** | **Question complete** |
| --- | --- | --- |
| 1. In an ideal world, what else would you add/ like to see? | - [In this ideal world] what do you see the role of a GP or other health practitioner in child mental health care? - [In this ideal world] where would you see MH services for children and families located? |  |
| 1. In this world, what is your role as a (*Paed/Psychiatrist/psychologist*) | 1. What is the role of a   *Paed/psychiatrist/psychologist* (other professions) |  |

**SECTION 3 – OTHER COMMENTS**

| 1. Is there anything else that would be helpful for us to know? |  |  |
| --- | --- | --- |

*[Optional/if applicable]* Is there anything about the system that surprises you?

*[We’ll provide everyone we interview with feedback about the findings of the project. Because it is a very large project we’ll be able to get back to you in about 12-18 months]*
